# Supplementary material for: Cross-Talks between Raf Kinase Inhibitor Protein and Programmed Cell Death Ligand 1 Expressions in Cancer: Role in Immune Evasion and Therapeutic Implications
Source: Cells. 2024 May 17;13(10):864. doi: 10.3390/cells13100864 (PMC11119125; doi:10.3390/cells13100864)
Supplement: Supplementary file 1 [file cells-13-00864-s001.zip › cells-2933799-supplementary.pdf]

Transcription Factors/ Motif For RKIP	chromosome	Start	Stop	BBLS	BLS	Z-score
HMG IY	chr12	118569963	118569970	2.753038	3.575322	3.747332
IRF-1	chr12	118574973	118574980	2.183653	3.092408	3.828954
LEF1	chr12	118575469	118575475	2.539901	3.018820	3.808140
MAFA	chr12	118574979	118574986	2.171181	3.144143	4.040388
MZF1	chr12	118574243	118574250	2.644240	3.393019	4.162756
**Transcription Factors/ Motif For PD-L1**	**chromosome**	**Start**	**Stop**	**BBLS**	**BLS**	**Z-score**
HMG IY	chr9	5447860	5447867	2.206903	2.235073	3.747332
IRF-1	chr9	5450334	5450341	3.131315	3.588257	3.828954
LEF1	chr9	5449945	5449951	3.041352	3.425722	3.808140
MAFA	chr9	5450056	5450063	1.885646	3.446501	4.040388
MZF1	chr9	5448402	5448409	1.944854	3.066747	4.162756

Supplementary Table 1: Similar motifs present on RKIP and PD-L1. This table provides the locations and starting/ending position in bp from the transcription start site (TSS) of the target gene or the closest gene. In addition, relative BBLS, BLS and Z-scores are provided. Z-score is the main indicator of the strength of predicted binding. It is specific to the binding motif (in PWM form) and the target sequence of the exact site. BBLS (Bayesian Branch Length Score), which is a measure of the conservation of the binding site – a score of 1 is significant while 4 is very strongly conserved.
